# The Effect of Magnet-to-Coil Distance on the Performance Characteristics of EMATs

**DOI:** 10.3390/s20185096

**Published:** 2020-09-07

**Authors:** Yutang Wu, Yunxin Wu

**Affiliations:** 1 School of Mechanical and Electrical Engineering, Central South University, Changsha 410083, China; wuyutang@csu.edu.cn; 2 State Key Laboratory of High-Performance Complex Manufacturing, Central South University, Changsha 410083, China; 3 Nonferrous Metal Oriented Advanced Structural Materials and manufacturing Cooperative Innovation Center, Central South University, Changsha 410083, China

**Keywords:** EMAT, lift-off effect, magnet-to-coil distance, conversion efficiency

## Abstract

The poor conversion efficiency and obvious lift-off effect of the electromagnetic acoustic transducer (EMAT) are commonly known to be problems for its practical application. For the purpose of enhancing the performance of EMATs, numerical simulations were performed in order to analyze the effect of various parameters. The results indicate that only the magnet-to-coil distance can effectively enhance the conversion efficiency and weaken the lift-off effect at the same time. When the magnet-to-coil distance is 2 mm, the lift-off effect will continue to be weakened as the magnet-to-coil distance increases, whereas the decrease of the lift-off effect is inconspicuous and the conversion efficiency starts to decline at this time. Therefore, to get the best performance of this specific EMAT, the suitable magnet-to-coil distance is 2 mm. The experiment effectively verifies the improvement of EMATs with a magnet-to-coil distance of 2 mm.

## 1. Introduction

Electromagnetic acoustic transducers (EMATs) are widely used in many industries. Like an eddy current sensor, which can provide abundant information [1,2,3,4,5], an EMAT can also be used to evaluate the property of materials and detect defects. Compared with piezoelectric transducers, EMATs can generate various modes of ultrasonic waves at the surface of the specimen without a couplant for a wholly different mechanism. However, the conversion efficiency is relatively low [6,7,8,9,10]. Previous research mainly focused on how to enhance the conversion efficiency, and the researchers achieved this goal by optimizing the magnet, coil, back-plate configuration, and so on [11,12,13]. In fact, not only the conversion efficiency but also the lift-off effect hinders the development of EMATs. Lift-off has a significant impact on the amplitude of the receiving signal of EMATs. A slight increase in the lift-off will cause obvious signal attenuation [14,15,16]. Therefore, in some specific work environments, for example, involving irregular sample surfaces, online inspection, and uneven coating, the signals are quite weak [17,18]. Weakening the lift-off effect can effectively improve this situation. However, studies on how to reduce the lift-off effect of EMATs are rarely reported.

The ultrasonic waves generated at the surface of the specimen are only induced by Lorentz force when the EMATs are operated on aluminum specimens [19,20,21]. The Lorentz force is determined by the magnetic flux density and the induced eddy current density at the specimen surface. Therefore, the conversion efficiency of EMATs is generally enhanced by improving these two physical quantities [11,12,13]. In this work, the magnet-to-coil distance, a parameter that has not received much attention, is studied in order to enhance the conversion efficiency of EMATs. A 2-D finite element model is developed to study the effect of magnet-to-coil distance on the magnetic flux density and the induced eddy current density at the specimen surface. Simulation results show that increasing the magnet-to-coil distance by an appropriate amount will result in a stronger induced eddy current density and a weaker magnetic flux density. The enhancement of induced eddy current density by increasing the magnet-to-coil distance obviously outweighs the loss of the eddy current density. Therefore, the conversion efficiency is improved significantly. Additionally, the nonlinear relationship between lift-off and peak-to-peak amplitude of the signal is transformed into a linear relationship by utilizing the lift-off effect. The slope of the linear curves is used to evaluate the lift-off performance. This study indicates that increasing the magnet-to-coil distance can improve the lift-off performance, and the magnet-to-coil distance is the only parameter that can increase the conversion efficiency and the lift-off performance effectively at the same time. Finally, some experiments are carried out to verify the improvement of the new EMAT.

## 2. Theory

In this paper, the EMAT is operated on an aluminum specimen. Therefore, there exists neither magnetostriction force nor magnetization force. Only the Lorentz force mechanism has to be taken into consideration [22,23].

**Figure 1:**
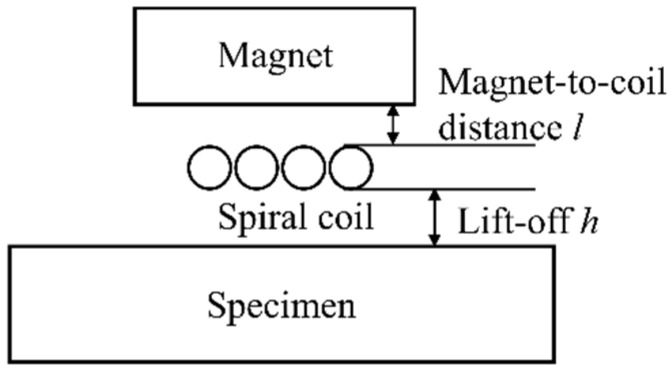
The configuration of a bulk wave electromagnetic acoustic transducer (EMAT).

A typical spiral coil EMAT is shown in Figure 1. This EMAT consists of a magnet, a spiral coil, and a piece of aluminum specimen. When an alternating current *I* is passing through the spiral coil, an eddy current field *J*_e_ within the skin depth of the specimen will be inducted. The induced eddy current *J*_e_ will periodically experience Lorentz force *F*, when the eddy current field *J*_e_ interacts with the magnetic field, as shown in Equation (3). The periodic vibration caused by the Lorentz force will produce ultrasonic waves whose propagation direction is perpendicular to the aluminum specimen surface. The induced eddy current *J_e_* is given by

(1) $$J_{e}=-\sigma \frac{\partial A}{\partial t}$$

where σ is the electrical conductivity of the specimen and *A* is the magnetic vector potential. The magnetic field’s control equation can be expressed as

(2) $$B=\mu_{0}\mu_{r}H+B_{r}$$

where *B* is the magnetic flux density, $\mu_{0}$ is the permeability of vacuum, $\mu_{r}$ is the relative permeability, *H* is the magnetic field intensity, and *B_r_* is the remanent magnetic flux density. The Lorentz force *F* is defined by

(3) $$F=J_{e}\times B$$

where *B* is the magnetic flux density. The balance equation of particle motion is

(4) $$\rho \frac{\partial^{2}u}{\partial t^{2}}=\nabla \cdot T+F$$

where ρ is the mass density of specimen, *u* is the elastic deformation, and *T* is the elastic stress tensor.

## 3. Results of Simulation and Discussion

### 3.1. Effect of Magnet-to-Coil Distance on Conversion Efficiency

**Figure 2:**
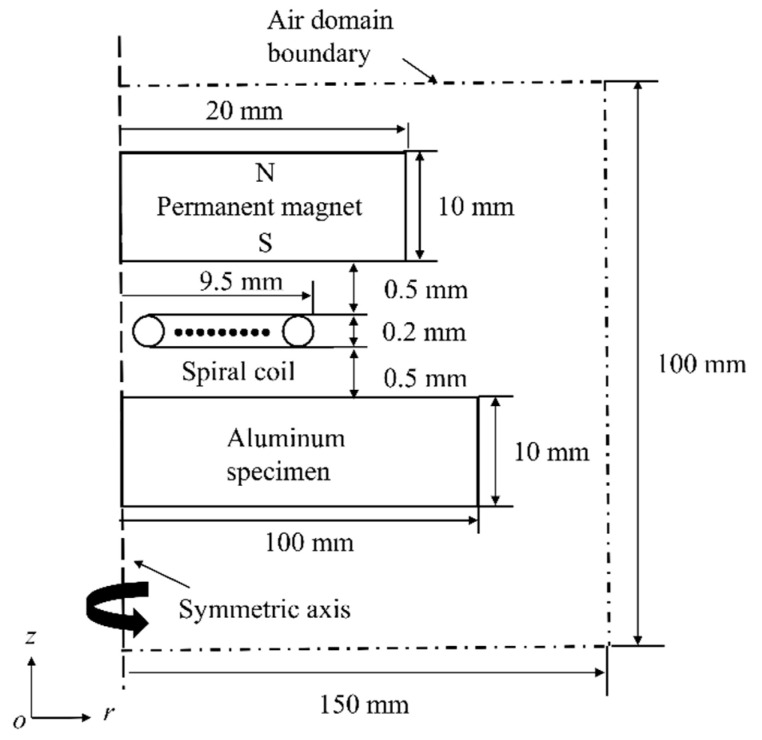
Geometrical schematic of the 2-D axis-symmetric model.

**Figure 3:**
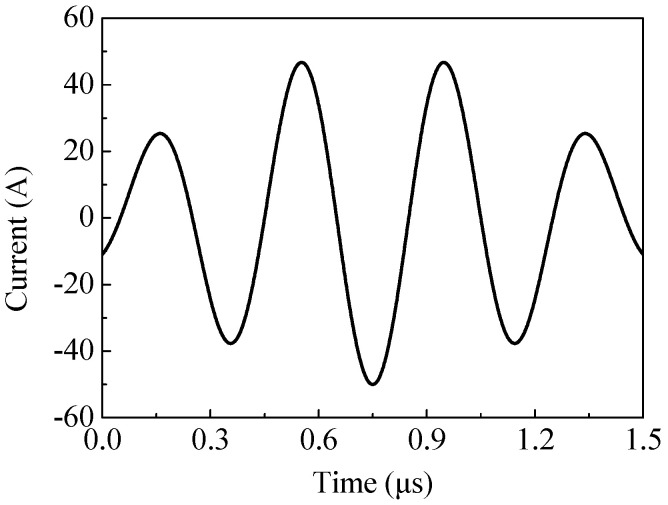
The excitation current in the simulation.

Optimizing structural parameters is one of the ways to improve conversion efficiency. Previous research has conducted detailed studies on the size of the magnet, as well as the size and shape of the coil and back-plate [11,12,13]. However, there exist few reports on the effect of magnet-to-coil distance on conversion efficiency. The magnet-to-coil distance will affect the magnetic flux density and the induced eddy current density at the specimen surface. Obviously, the magnetic flux density will decrease as the magnet-to-coil distance increases. As for induced eddy current density, when the magnet-to-coil distance increases, the induced eddy current density at the magnet surface will decrease. Correspondingly, the induced eddy current density at the specimen surface will increase. This is because the energy of the electrical signal *I* transfers to the specimen surface and the magnet surface near the coil. The key research question is whether the enhancement of induced eddy current density outweighs the loss in magnetic flux density. To solve this problem, a 2-D finite element model was developed. Due to the symmetries of this EMAT, a 2-D axis-symmetric model was utilized to calculate the numerical results. As shown in Figure 2, the *oz* axis is the symmetric axis, and the radius and the height of the permanent magnet are 20 mm and 10 mm, respectively. The radius and the height of the aluminum specimen are 100 mm and 10 mm, respectively. In addition, the EMAT is surrounded by air domain boundaries. Table 1 shows the basic parameters for a finite element model. The excitation current is shown in Figure 3. The peak of the excitation current is 50 A with a time range of 0–1.5 μs. The model was solved by COMSOL Multiphysics 5.2 (COMSOL Inc., Burlington, MA, USA). The AC/DC and Magnetic Field module was used for producing Lorentz force, and Structural Mechanics was used for the generation and transmission of shear waves.

**Figure 4:**
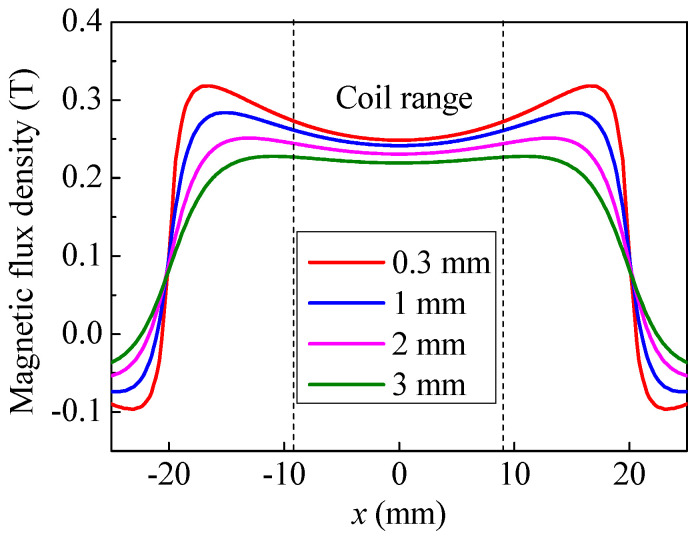
The magnetic flux density profile at the specimen surface.

Figure 4 shows the magnetic flux density at the specimen surface. In Figure 4, the magnetic flux density distribution has a flat arched curve at the center, and the magnetic flux density is slightly larger towards the edges of the magnet. If the magnet-to-coil distance increases, the magnet-to-specimen distance will increase as well. When the magnet-to-coil distance is increased from 0.3 mm to 3 mm, the maximum attenuation of magnetic flux density at the edge of the coil is 21.1% while the minimum attenuation at the center of coil is 13.3%. In addition, if the magnet-to-coil distance is increased from 1 mm to 2 mm, and 2 mm to 3 mm, the maximum attenuation is 7.33% and 4.99%, respectively. Therefore, the attenuation trend of magnetic flux density becomes more obvious as the magnet-to-coil distance increases.

**Figure 5:**
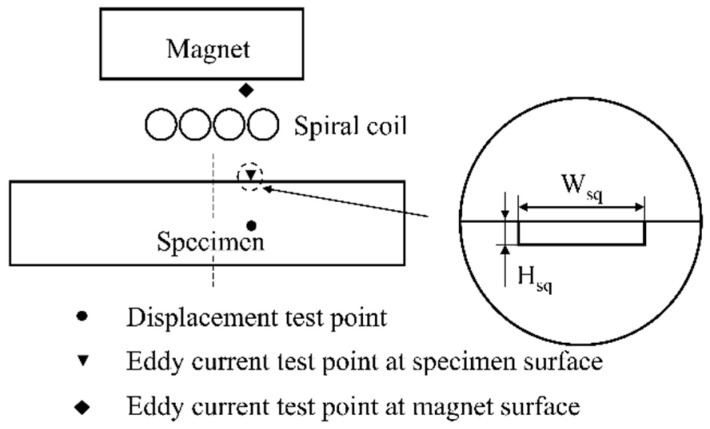
The eddy current and the displacement test point.

**Figure 6:**
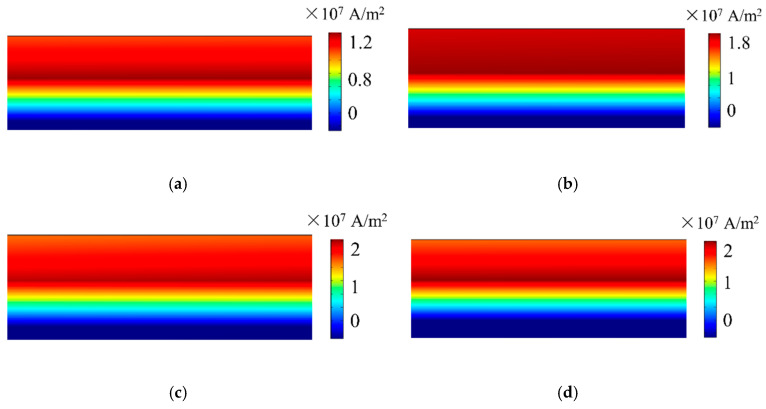
The eddy current distribution in the specimen at 8 μs generated by the EMAT with a magnet-to-coil distance of (**a**) 0.3 mm, (**b**) 1 mm, (**c**) 2 mm, and (**d**) 3 mm.

**Figure 7:**
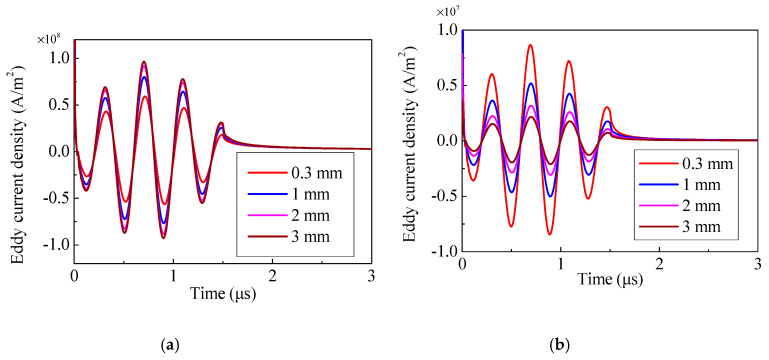
The eddy current density at the test point on the surface of (**a**) the specimen and (**b**) the magnet.

An eddy current field will be generated at the surface of specimen when the specimen interacts with the alternating current in the coil. Because the standard depth of penetration of the current is quite shallow, two points and a small square domain are set up to observe the current conveniently. The points are 5 mm away from the center of the specimen, and the width and height of the small square domain are 1.8 mm and 0.7 mm, respectively, as shown in Figure 5. The eddy current distribution of different magnet-to-coil distances is shown in Figure 6. The eddy current is mainly distributed in a shallow surface layer for the skin effect. Additionally, it can be seen that as the distance increases, the eddy current density increases as well. To investigate the behavior of eddy current density under various magnet-to-coil distances, the eddy current density at the test point on the specimen surface was calculated and is shown in Figure 7a. As the magnet-to-coil distance increases, the current density at the specimen surface increases as well. The reason for this is that the energy transferred to the magnet is decreased when the magnet-to-coil distance increases, as is shown in Figure 7b. In addition, the increase in current density at the specimen surface and the decrease in current density at the magnet surface gradually diminish. Taking current density at the specimen surface as an example, when the magnet-to-coil distance is increased from 1 mm to 2 mm, the increase in current density is 14.4%. However, when the distance is increased from 2 mm to 3 mm, the increase drops to 5.26%, which leads to the almost overlapping of the two corresponding curves in Figure 7a. Incorporating the above analysis, the conversion efficiency of an EMAT cannot be enhanced continuously by increasing the magnet-to-coil distance.

**Figure 8:**
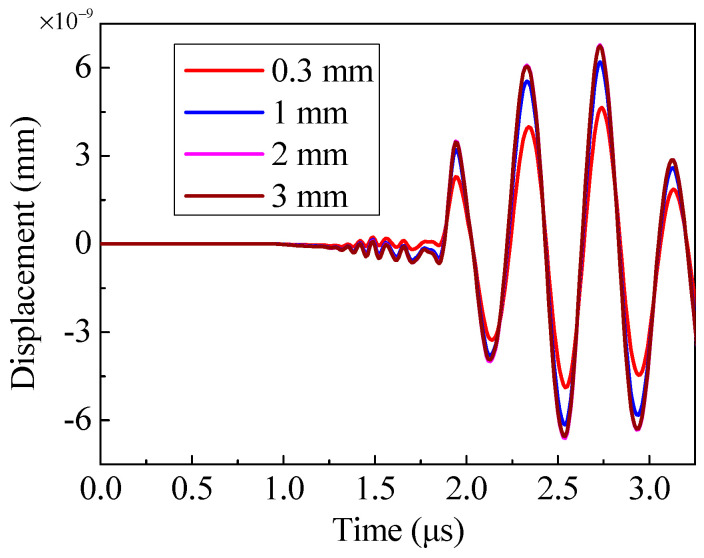
The shear waves generated by different configurations.

**Figure 9:**
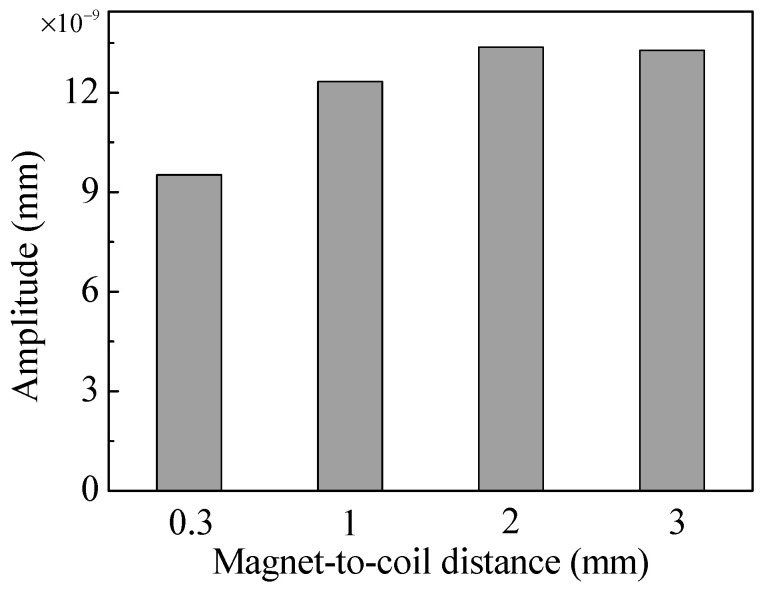
The peak-to-peak amplitude of shear waves.

Based on the trends of the magnetic flux density and eddy current density, it is reasonable to assume that the amplitude of the shear waves generated by the EMAT will increase first and then decrease slowly as the magnet-to-coil distance increases. The waveforms at the displacement test point in Figure 8 confirm this hypothesis. Note that the curves of 2 mm and 3 mm almost overlap in Figure 8. To conveniently contrast the amplitude of the shear wave, the peak-to-peak amplitude of the waves is plotted in Figure 9. The conversion efficiency in the EMAT with a magnet-to-coil distance of 2 mm is about 1.4 times as high as that in the EMAT with a magnet-to-coil distance of 0.3 mm. That is, increasing the magnet-to-coil distance can significantly enhance the conversion efficiency of EMAT; however, when the magnet-to-coil distance reaches 2 mm, the amplitude of the shear wave starts to decrease slowly. Therefore, the magnet-to-coil distance should be 2 mm to get the best conversion efficiency.

### 3.2. Influence of Magnet-to-Coil Distance on Lift-Off Effect

The lift-off effect can easily affect the amplitude of signals generated by EMATs, which may result in signals being masked by noise. Research on weakening the lift-off effect of EMATs has been rarely reported. Moreover, there exists no feature that can be used to evaluate the lift-off performance.

The amplitude will reduce exponentially when the lift-off increases, which was confirmed in the research by Ogi et al. and Thompson [15,24]. Hence, the relationship between lift-off and signal amplitude can be expressed as

(5) $$A_{s}=\;e^{k\cdot h+b}$$

where *A*_s_ is the signal amplitude, *h* is the lift-off, and b and k are constants related to the configuration. Taking the logarithm of both sides of Equation (5), a new equation is obtained:

(6) $$\ln\left(A_{s}\right)=\;k\cdot h+b$$

By treating ln(*A*_s_) as a dependent variable, Equation (6) can be regarded as a linear equation. The value of k will not change with the lift-off. Therefore, the value of k can be used to describe the decay rate of the signal. In other words, the lift-off performance can be characterized by feature k. If the function graph of the linear equation becomes smoother as the lift-off changes, that is, the smaller the slope becomes, the better the lift-off performance is.

**Figure 10:**
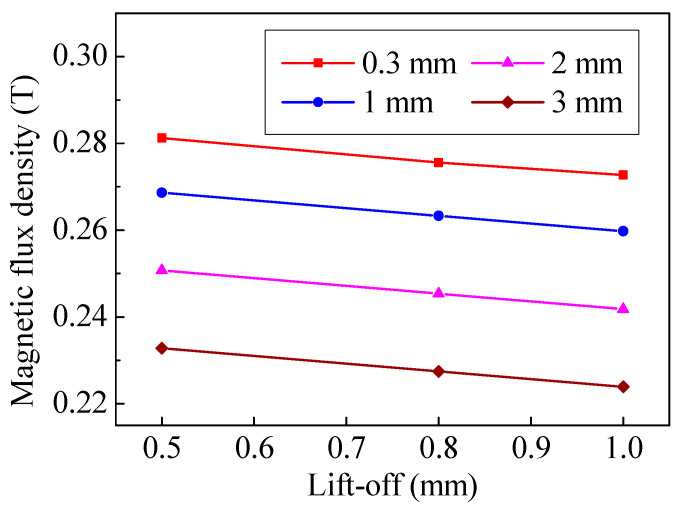
The relationship between magnetic flux density and lift-off.

**Figure 11:**
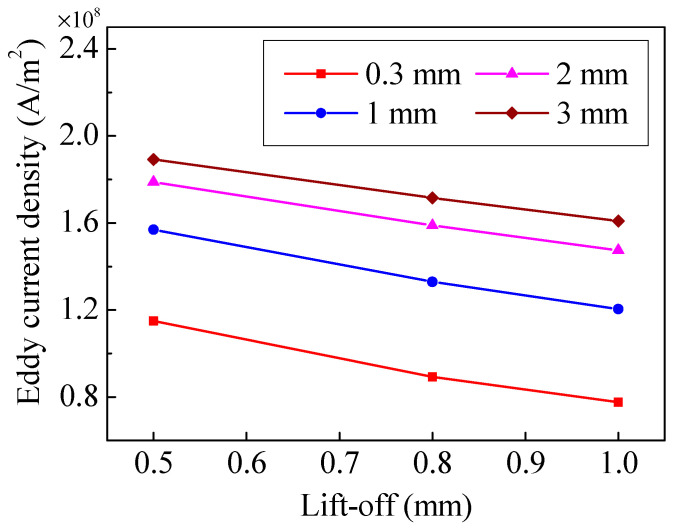
The relationship between eddy current density and lift-off.

Obviously, as one of the main factors that can directly affect the amplitude of ultrasonic waves, the distribution of Lorentz force at the specimen is sensitive to lift-off. To conduct an in-depth study on the effect of magnet-to-coil distance on lift-off effect, the effect of lift-off on magnetic flux density and eddy current density were investigated. Figure 10 shows the relationship between lift-off and magnetic flux density at the edge of the coil. It can be observed that the magnetic flux density is related to the lift-off. The magnetic flux density decreases with increase of lift-off. In the previous section, it was concluded that the attenuation trend of magnetic flux density becomes more obvious with a greater magnetic-to-coil distance. However, the biggest distance difference in lift-offs is only 0.5 mm in Figure 10, which leads to almost the same attenuation trend of magnetic flux density. On the other hand, the eddy current density decreases with lift-off as well, which is shown in Figure 11. In Figure 11, it is obvious that a greater lift-off means a slower decay rate in eddy current density. When the magnet-to-coil distance is 0.3 mm, if the lift-off is increased from 0.5 mm to 1 mm, the decay rate of the eddy current density is 35.2%. Once the magnet-to-coil distance is 2 mm and 3 mm, the decay rate of the eddy current density drops to 17.4% and 15.3%, respectively. Therefore, increasing the magnet-to-coil distance can limit the eddy current density attenuation.

**Figure 12:**
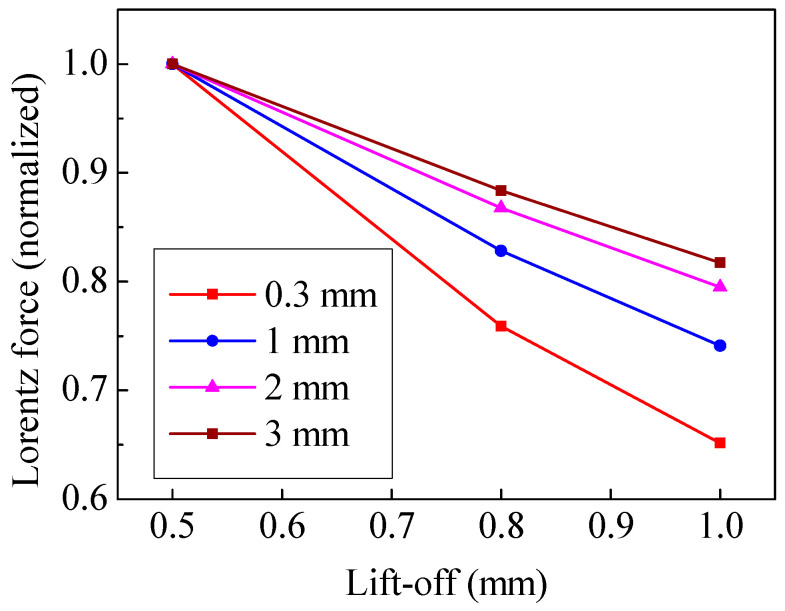
The relation of lift-off and Lorentz force.

**Figure 13:**
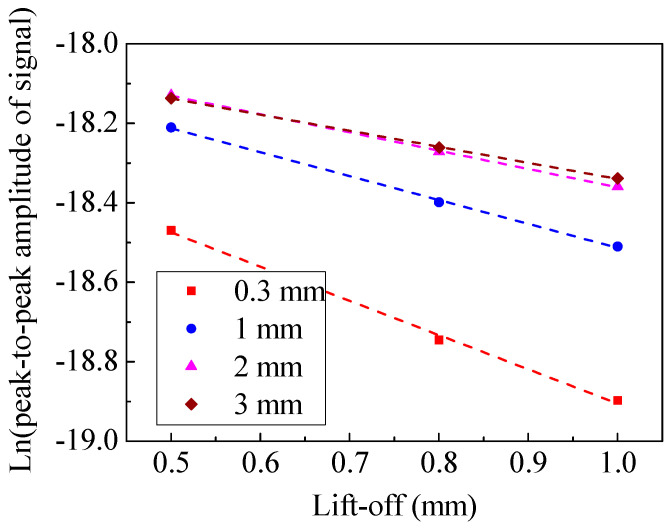
The relationship between lift-off and ln(*A*_s_).

The Lorentz force is determined by the magnetic flux density and the eddy current density at the specimen surface. Therefore, the trend of Lorentz force is similar to that of the eddy current density, as shown in Figure 12. It can be seen that increasing the magnet-to-coil distance is conducive to a slower decay rate of the Lorentz force. The relationship between lift-off and ln(*A*_s_) is shown in Figure 13. The slope of the fitting lines in Figure 13 indicates that a larger magnet-to-coil distance means better lift-off performance. Table 2 shows the k value and correlation coefficient of different EMATs. It can be observed that when the magnet-to-coil distance is increased from 0.3 mm to 2 mm, the lift-off performance is increased by 46.6%. However, when the magnet-to-coil distance is increased from 2 mm to 3 mm, the lift-off performance is only increased by 12.2%. Therefore, the promotion of lift-off performance is diminishing as the magnet-to-coil distance increases, which demonstrates that the ability to improve the lift-off performance by increasing the magnet-to-coil distance is limited. In addition, the correlation coefficients in Table 2 are quite close to −1, which indicates that the simulation results are reliable. All in all, increasing the magnet-to-coil distance can weaken the lift-off effect.

For an EMAT, a good lift-off performance and high conversion efficiency are indispensable. This implies that the position of the fitting line of an excellent EMAT should be as high as possible in Figure 13. Meanwhile, the trend of the fitting line should be as smooth as possible. The above requirements can be achieved by appropriately increasing the magnet-to-coil distance. When the magnet-to-coil distance is 2 mm, the lift-off performance is increased by 46.6%. Incidentally, the conversion efficiency is increased by 40.5% and 71.0% with a lift-off of 0.5 mm and 1 mm, respectively.

**Figure 14:**
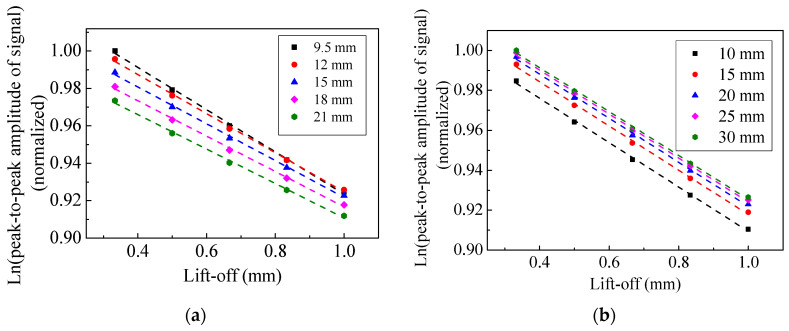
The effect of (**a**) magnet radius and (**b**) magnet height on the performance of an EMAT.

In fact, the magnet-to-coil distance is the only parameter that can weaken the lift-off effect and enhance the conversion efficiency at the same time. The diameter of wire, magnet size, frequency of excitation current, remanent flux density, and so on can only improve either the lift-off performance or the conversion efficiency. Due to space constraints, Figure 14 only shows the effect of magnet size on the performance of an EMAT, in which it can be observed that increasing magnet height and decreasing magnet radius are beneficial to conversion efficiency. However, the lift-off performance drops as the conversion efficiency increases.

When the magnet-to-coil distance is 2 mm, the lift-off performance will continue to be improved as the magnet-to-coil distance increases. However, the conversion efficiency starts to fall. Therefore, considering both the conversion efficiency and the lift-off performance, the magnet-to-coil distance should be 2 mm in order to get the best performance of this EMAT.

## 4. Experimental Validation

**Figure 15:**
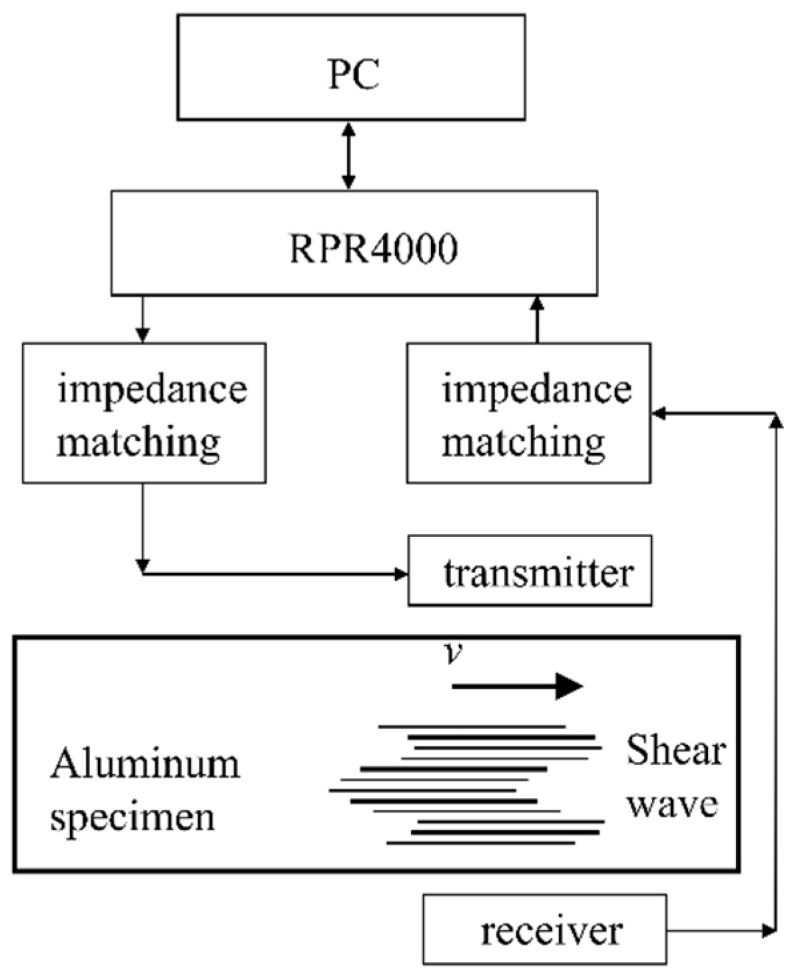
The configuration of experimental setup.

To verify the effect of magnet-to-coil distance on the performance of an EMAT, two groups of experiments were carried out. The experimental setup is shown in Figure 15. The whole system worked in a pitch-catch configuration. The first group measured the improvement of the conversion efficiency of an EMAT with suitable lift-off, and the second compared the effect of magnet-to-coil distance on lift-off performance.

The configuration of the EMATs was the same as that in the finite element model in Section 3. A spiral coil EMAT was used to receive the signal generated by the EMATs with different magnet-to-coil distances. The RPR4000 was configured to excite the coil in the transmitter EMAT and receive the electric signal in the receiver EMAT. The function of impedance matching was to match the impedance of coils. The distance between two EMATs remained unchanged. The lift-off and magnet-to-coil distance were ensured by B5 paper, as the thickness of a sheet of B5 paper is 0.1 mm. In the next experiments, the lift-off of the transmitter EMAT was adjusted to 0.5 mm, 0.7 mm, 0.8 mm, and 1 mm. The least squares method was utilized to fit the lift-off and ln(*A*_s_).

**Figure 16:**
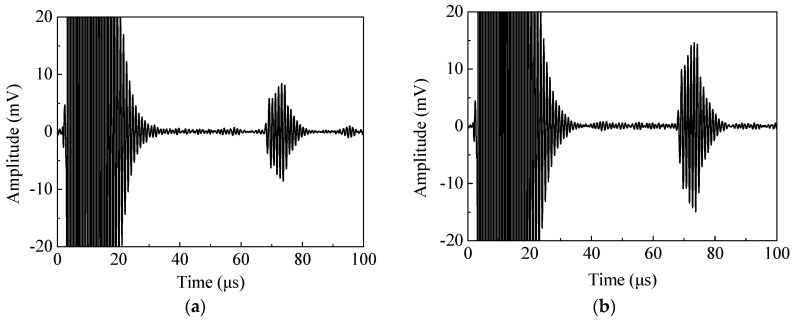
The received signal with (**a**) a magnet-to-coil distance of 0.3 mm and (**b**) a magnet-to-coil distance of 2 mm.

**Figure 17:**
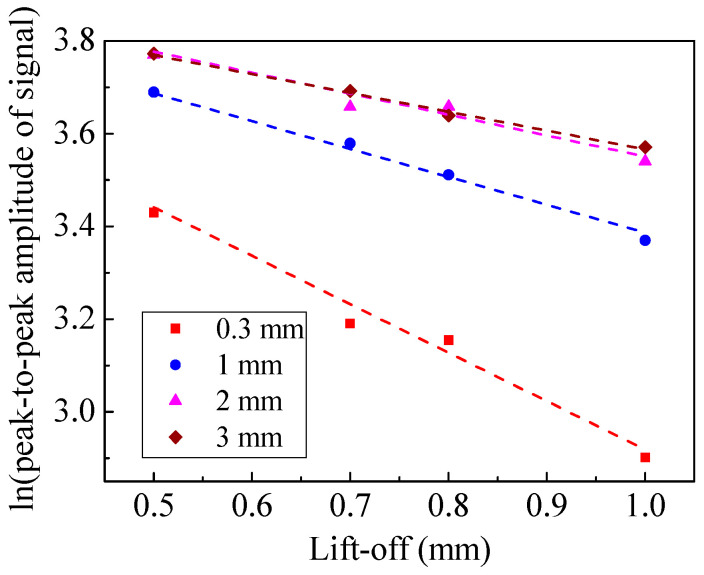
The effect of magnet-to-coil distance on the performance of an EMAT.

Figure 16 shows the measured shear waveforms with different magnet-to-coil distances when the lift-off was 1 mm. The amplitude peak in Figure 16b is obviously larger than that of Figure 16a. The generation efficiency of the EMAT was increased by 67.5%. This value is close to the numerical simulation results, showing a significant improvement in the generation efficiency of an EMAT with a suitable magnet-to-coil distance. In addition, the effect of magnet-to-coil distance on performance of EMAT is shown in Figure 17. In Figure 17, it can be observed that a suitable magnet-to-coil distance can significantly enhance the conversion efficiency and the lift-off performance at the same time. This means that increasing the magnet-to-coil distance appropriately can enhance the eddy current density at the specimen surface and limit the eddy current density attenuation with increasing lift-off.

## 5. Conclusions

The poor conversion efficiency is one of the main obstacles hindering the development of electromagnetic acoustic techniques. In this paper, an in-depth study of magnet-to-coil distance on conversion efficiency of EMAT is conducted. The magnet-to-coil distance will affect the magnetic flux density and eddy current density at the specimen surface. The results of the study indicate that properly increasing the distance can increase the current significantly and reduce the magnetic flux density weakly. When the lift-off is 0.5 mm, if the magnet-to-coil distance increases from 0.3 mm to 2 mm, the maximum drop in the magnetic flux density is 21.1%, while the increment of eddy current density is 54.7%. Therefore, the enhancement of induced eddy current density outweighs the loss in magnetic flux density, which results in a 40.5% conversion efficiency growth. What’s more, the conversion efficiency growth reaches 71.0% when the lift-off is 1 mm.

In some special circumstances, in addition to a high conversion efficiency, a good lift-off performance of an EMAT is required as well. The numeric simulation suggests that a great magnet-to-coil distance can effectively limit the eddy current density attenuation with increasing lift-off. Meanwhile, the attenuation of the magnetic flux density remains almost unchanged. In this paper, the nonlinear relationship between lift-off and receiving signal amplitude is transformed into a linear relationship. Hence the lift-off performance of an EMAT can be represented by the value of slope k of the fitting line. The results showed that when the magnet-to-coil distance increases, the corresponding Lorentz force decays more slowly as the lift-off increases. That is, the lift-off performance is optimized. Specifically, when the magnet-to-coil distance is 2 mm, the lift-off performance increases by 46.6%. Continuing to increase the distance can slightly improve the lift-off performance at this time. Furthermore, the conversion efficiency starts to decrease at the same time. Therefore, the magnet-to-coil distance should be 2 mm in order to ensure the greatest performance improvement of this specific EMAT.

Some experiments were carried out to validate the improvement of an EMAT with a proper magnet-to-coil distance. The experimental results supported the numerical simulation. The results demonstrated that an EMAT with a magnet-to-coil distance of 2 mm can enhance the conversion efficiency and weaken lift-off performance significantly at the same time.

## Figures and Tables

**Table 1:** The parameters for creating the EMAT model.

Description, Symbol	Value, Unit
Remanent flux density, B_r_	1.2 T
Magnet size	20 mm × 10 mm
Aluminum specimen size	100 mm × 10 mm
Lift-off	0.5 mm
Electrical conductivity, Al	3.774 × 10^7^ s/m
Electrical conductivity, NdFeB Magnet	6.25 × 10^5^ s/m
Electrical conductivity, coil	2.667 × 10^7^ s/m
Excitation current frequency	2.5 MHz

**Table 2:** The slope of the curve (*h*, ln(*A*_s_)) and the correlation coefficient.

Magnet-to-Coil Distance (mm)	k	Correlation Coefficient
0.3	−0.8615	−0.9986
1	−0.6016	−0.9990
2	−0.4603	−0.9996
3	−0.4041	−0.9998
